# Mechanisms of rapid cancer cell reprogramming initiated by targeted receptor tyrosine kinase inhibitors and inherent therapeutic vulnerabilities

**DOI:** 10.1186/s12943-018-0816-y

**Published:** 2018-02-19

**Authors:** Emily K. Kleczko, Lynn E. Heasley

**Affiliations:** 1 Division of Renal Diseases and Hypertension, Department of Medicine, School of Medicine, University of Colorado Anschutz Medical Campus, Aurora, CO 80045 USA; 2 Department of Craniofacial Biology, School of Dental Medicine, University of Colorado Anschutz Medical Campus, Aurora, CO 80045 USA

## Abstract

Receptor tyrosine kinase (RTK) pathways serve as frequent oncogene drivers in solid cancers and small molecule and antibody-based inhibitors have been developed as targeted therapeutics for many of these oncogenic RTKs. In general, these drugs, when delivered as single agents in a manner consistent with the principles of precision medicine, induce tumor shrinkage but rarely complete tumor elimination. Moreover, acquired resistance of treated tumors is nearly invariant such that monotherapy strategies with targeted RTK drugs fail to provide long-term control or cures. The mechanisms mediating acquired resistance in tumors at progression treated with RTK inhibitors are relatively well defined compared to the molecular and cellular understanding of the cancer cells that persist early on therapy. We and others propose that these persisting cancer cells, termed “residual disease”, provide the reservoir from which acquired resistance eventually emerges. Herein, we will review the literature that describes rapid reprogramming induced upon inhibition of oncogenic RTKs in cancer cells as a mechanism by which cancer cells persist to yield residual disease and consider strategies for disrupting these intrinsic responses for future therapeutic gain.

## Background

Receptor tyrosine kinases (RTKs) function as oncogene drivers in solid tumors through diverse mechanisms including mutation, amplification and autocrine/paracrine activation. As an example, lung adenocarcinomas (LUADs) harbor diverse oncogenic RTKs and many, such as EGFR, ALK, and ROS1 have approved tyrosine kinase inhibitors (TKIs) that induce dramatic tumor responses [1–8]. Additional oncogenic drivers such as MET, RET and NTRK1 have more recently emerged and promising TKIs are under development [9–12]. EGFR activation in head and neck squamous cell carcinoma (HNSCC) through overexpression and autocrine/paracrine mechanisms is frequent and antibody-based EGFR inhibitors such as cetuximab are approved therapeutics that provide benefit [13–16]. Similarly, the ERBB2 oncogene is amplified or overexpressed in ~ 20% of breast cancers and inhibitory ERBB2 antibodies are effective in the treatment of these cancers [17]. While application of precision medicine with therapeutics targeting RTKs yields dramatic responses in LUADs bearing oncogenic EGFR, ALK and ROS1 [1–4, 6–8], chronic control or cures have not yet been realized due to the inevitability of acquired resistance leading to tumor relapse. Likewise, treatment failures to EGFR and ERBB2 therapies in HNSCC and breast cancer are associated with frequent acquired resistance.

Over the past decade, mechanisms mediating acquired resistance to RTK inhibitors have been investigated through analysis of tumor samples obtained at disease progression and represents a thoroughly reviewed topic [18–20]. Selection for acquired mutations that prevent TKI binding are frequent and next-generation inhibitors have been developed to block the drug-resistant forms of the oncogenic RTK. For example, the 3rd generation EGFR inhibitor, osimertinib, effectively inhibits the EGFR-T790 M protein that emerges in response to treatment with 1st generation EGFR inhibitors [21]. While subsequent clinical responses to osimertinib can be striking, tumor elimination is still incomplete and is eventually accompanied by tumor progression. Thus, acquired resistance to targeted therapeutics likely represents the outgrowth of evolutionarily dominant clones and has encouraged a strategy of reacting to resistance rather than primary prevention. Similar to the experience with early therapeutic strategies for HIV or tuberculosis (reviewed in [22]), strategies involving the deployment of sequential monotherapies, even with 2nd and 3rd generation agents, seem unlikely to yield long-term cancer control or cures.

A common feature of treatment failure with monotherapy, whether antimicrobial or anticancer, is the incomplete elimination of the bacterial or tumor cell targets [22, 23]. These persisting bacteria or cancer cells survive without evidence of mutations conferring drug resistance and with regard to cancer, have been referred to as “drug tolerant persisters” [24] or “residual disease” [23]. In addition to the concept reviewed herein that tumor cell reprogramming provides a mechanism for residual disease, the literature also supports intrinsic resistance of subsets of tumor cells due to intratumoral heterogeneity (see [25] for an example relevant to lung cancer). Alternatively, pharmacokinetic failure can also provide a mechanism for incomplete tumor cell elimination. Central to this review article is the premise that residual disease, even after highly effective treatment with oncogene-targeted drugs, is responsible for eventual relapse. As a TKI-relevant example, analysis of the degree of tumor shrinkage in response to ALK inhibitors in patients with EML4-ALK positive lung cancer revealed a highly significant positive correlation with overall and progression-free survival [26]. We propose that persisting tumor cells represent the major hurdle to further increases in the efficacy of targeted cancer therapies by serving as an incubator for eventual emergence of tumor cell clones that are resistant to the TKI. Thus, therapeutic regimens that achieve chronic, durable or curative goals must effectively eliminate this reservoir of residual disease. Herein, we will review the literature that supports the role of rapid tumor cell reprogramming as a mechanism promoting survival of persisting cancer cells following RTK-targeting agents through cancer cell autonomous and non-autonomous pathways involving paracrine communication with the tumor microenvironment (TME). Finally, we will consider rational combination strategies that might be deployed to eliminate or minimize residual disease.

### RTK inhibitor-induced reprogramming with tumor cell autonomous functions

#### Rapidly induced bypass pathways

**Fig. 1:**
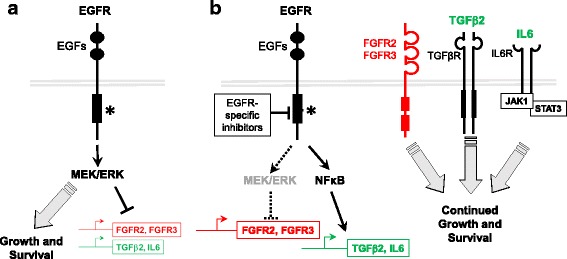
Rapidly induced cell autonomous bypass signaling. **a** Using EGFR mutated lung tumors as an example, oncogenic EGFR signals through the MEK/ERK pathway to drive growth and survival, but also suppresses FGFR2, FGFR3 and TGFβ2 expression. **b** Upon treatment with EGFR-specific TKIs, MEK/ERK activity is inhibited to reduce growth and survival signaling, but also de-represses alternative bypass growth and survival pathways including FGFR2, FGFR3, TGFβ2 and IL6. EGFR TKIs increase NFκB pathway activity which may drive expression of IL6 [36]. These transcriptional changes result in establishment of emergent autocrine loops to ensure the continued growth and survival of the tumor in the presence of an EGFR inhibitor

An extensive literature demonstrates the dynamic nature of the kinome, the subset of the genome encoding protein kinases [27], in response to drugs that inhibit dominant oncogenic pathways in cancer cells (reviewed in [28–31]). It is not our intention to exhaustively re-visit this literature here, but to highlight several recurring themes where rapid reprogramming may support tumor cell persistence in RTK-driven cancers (see Fig. 1). A number of studies support the ability of TKIs to promote rapid de-repression of distinct RTKs, thereby providing emergent growth and survival signaling to bypass the inhibited receptor. In fact, the degree to which oncogene targeted agents lead to increased gene expression is relatively unappreciated compared to reduced gene expression events. Ware et al. [32] demonstrated rapid induction of fibroblast growth factor receptor (FGFR) 2 and FGFR3 expression in EGFR-dependent lung cancer cells treated with EGFR-specific TKIs and cetuximab. FGFR2 induction was also induced by SRC and mitogen-activated protein kinase kinase (MAP2K, MEK) inhibitors, suggesting that these pathways may mediate EGFR-dependent repression of FGFR2 and FGFR3. In support, Sharifnia et al. [33] deployed an ORF-based kinase screen to identify potential bypass signaling pathways in EGFR mutant PC9 lung cancer cells and identified both FGFR2 and FGFR1 (see below). In a glioblastoma cell line [34], EGFR-specific TKIs transcriptionally de-repressed platelet-derived growth factor receptor β (PDGFRβ). The data supported a mechanism whereby EGFRvIII signaling actively suppresses PDGFRβ transcription in a target of rapamycin complex 1 (TORC1)- and extracellular signal–regulated kinase (ERK)-dependent manner. A distinct study in EGFR mutant lung cancer cell lines demonstrated that EGFR-specific TKIs engage a positive feedback loop involving induction of FGFRs and IL6, leading to STAT3 activation to promote cell survival and limit overall drug-induced growth inhibition [35]. Specifically, MAP2K/MEK inhibition led to autocrine activation of STAT3 via FGFR2, FGFR3 and, distal to IL6 and its receptor, Janus kinases (JAKs). Inhibition of MEK together with JAK and FGFRs enhanced tumor xenograft regression. Also in EGFR mutant lung cancer cell lines, NFκB signaling was found to be rapidly induced upon EGFR inhibitor treatment to promote tumor cell survival and residual disease [36]. Mechanistically, inhibition of oncogenic EGFR induced the formation of an EGFR-TRAF2-RIP1-IKK complex that stimulated NFκB-dependent transcription including increased IL6 which functioned in an autocrine fashion to stimulate STAT3 and survival. Combined, these studies support the involvement of the MEK/ERK pathway in kinome reprogramming as well as NFκB signaling proximal to IL6 expression.

Klezcko et al. [37] analyzed gene expression changes in HNSCC cell lines treated for 3 days with TKIs targeting EGFR and/or FGFRs and identified transforming growth factor beta 2 (TGFβ2) as a rapidly and broadly induced gene. TGFβ2 mRNA was also increased in patient-derived HNSCC xenografts treated with cetuximab, demonstrating in vivo relevance of these findings. Moreover, functional genomics screens identified TGFβ2 and TGFβ receptors (TGFβRs) as synthetic lethal genes in the context of TKI treatment. Direct RNAi-mediated silencing of TGFβ2 and pharmacological TGFβR inhibitors reduced cell growth, both alone and in combination with TKIs. In summary, the studies support a TGFβ2-TGFβR pathway as a TKI-inducible growth pathway in HNSCC that limits efficacy of EGFR-specific inhibitors.

The literature reveals that altered gene transcription is not required for all rapid, TKI-induced reprogramming. Zhang et al. [38] used phospho-tyrosine affinity purification coupled with mass spectrometry in an EML4-ALK fusion oncogene-driven lung cancer cell line, H3122, to define an ALK signaling network. Among the network elements identified, tyrosine phosphorylation of EGFR was shown to be increased upon treatment with ALK inhibitors. In a distinct study, Vaishnavi et al. [39] specifically investigated the signaling requirement for EGFR in a panel of lung cancer cell lines driven by oncogenic fusion kinases including ALK, but also ROS1, NTRK1 and RET. Interestingly, fusion kinase inhibition enhanced binding of adaptor proteins to EGFR to yield continued signaling in the setting of TKI treatment. These findings unveil how non-mutated EGFR can provide rapid, transcription-independent adaptive survival signaling and cancer cell persistence in the setting of oncogene-specific inhibitors. Consistent with these findings, Singleton et al. [40] deployed a genome-wide RNAi screen to identify genes whose silencing potentiate the inhibitory effect of FGFR-specific TKIs in HNSCC cell lines. The results revealed a role for multiple RTKs including EGFR, ERBB2 and MET in maintaining growth and survival signaling in HNSCC cells in the setting of FGFR inhibition. Moreover, triple combinations of TKIs inhibiting FGFRs, EGFR/ERBB2 and MET yielded greater growth inhibition compared to any double combination. As a group, these studies provide support for the concept of RTK co-activation networks in cancer cells [31, 41] and suggest that the inherent signaling flexibility provides a mechanism for incomplete growth inhibition with single TKI treatments.

#### RTK signaling alterations with cell phenotype changes

**Fig. 2:**
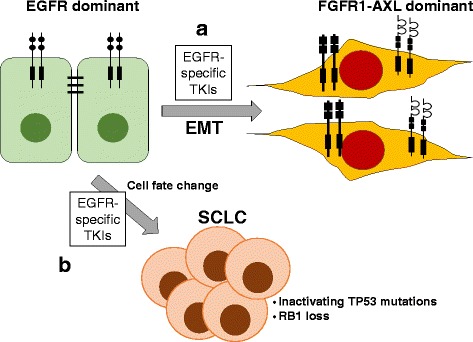
Phenotypic changes in EGFR mutant LUAD as a mechanism of resistance to targeted therapy. **a** In response to EGFR inhibitors, multiple groups have observed that EGFR mutant LUAD can undergo an epithelial to mesenchymal transition (EMT). Cells that have undergone EMT no longer rely on oncogenic EGFR as a driver, but rather on FGF2-FGFR1 and AXL signaling pathways induced as a consequence of EMT. **b** Clinically, EGFR mutant LUADs treated with EGFR-specific TKIs undergo a phenotypic switch to small cell lung cancer (SCLC) accompanied by TP53 mutant and loss of RB1 expression. This cell fate change has not been observed with in vitro models and the kinetics and mechanism are not well understood

In addition to mechanisms involving rapidly induced bypass signaling, RTK inhibitor-induced epithelial to mesenchymal transition (EMT) has emerged as a mechanism of resistance, especially in response to EGFR inhibitors. EMT (reviewed in [42]) is an important consideration in the setting of residual disease since marked switching in RTK pathway dominance has been shown to occur (Fig. 2). Multiple groups have submitted EGFR mutant lung cancer cell lines to in vitro selection procedures with EGFR-specific TKIs and noted an increase in mesenchymal differentiation in the resulting TKI-resistant cultures [43–46]. Furthermore, this mechanism of resistance is not unique to lung cancer as an EMT mechanism of resistance to EGFR inhibition has been observed in HNSCC cell lines [47, 48]. As a rule, EGFR-dependent cancer cell lines that have undergone EMT as a mechanism of acquired resistance fail to exhibit previously documented molecular events such as selection for the EGFR T790 M gate-keeper mutation or MET amplification [18]. Considering the marked difference in RTK dominance in isogenic epithelial and mesenchymal pairs of lung cancer cell lines generated through in vitro acquired TKI resistance [49, 50], it is likely that TKI insensitivity in these models is related to the emergence of distinct RTK pathways as growth drivers including FGFR family members and AXL. Ware et al. used multiple EGFR mutant lung cancer cell lines rendered EGFR TKI resistant to demonstrate that acquisition of a mesenchymal phenotype was associated with acquired addiction to an FGF2-FGFR1 autocrine loop [45]. This switch in pathway dependency was mediated by increased expression, but not amplification, of FGF2 and FGFR1. Moreover, growth of the resistant cultures could be completely inhibited by distinct FGFR-specific TKIs. While AXL was also increased in the EGFR TKI-resistant cell lines, growth sensitivity to crizotinib was not observed. However, in other studies, induction of a Gas6-AXL pathway associated with EMT was shown to mediate EGFR TKI-induced resistance in lung cancer [51, 52] to cetuximab and erlotinib in HNSCC [53, 54] and to ALK inhibitors in ALK^F1174L^-positive human neuroblastoma cells [55].

Because of the chronic method by which TKI-resistant cell lines exhibiting mesenchymal differentiation were selected, it is unclear if TKI-induced EMT represents a rapid reprogramming event. Anecdotally, cell morphology changes occur within weeks of TKI treatment [45]. Also, our own data from RNAseq analysis of EGFR mutant HCC4006 LUAD cells treated with EGFR TKIs over a time course of hours to weeks reveal loss of CDH1 and ESRP1, epithelial markers, within a week followed by more progressive induction of mesenchymal genes within 2 to 3 weeks [56]. Thus, it seems plausible that therapy-induced EMT could emerge within the time frame of initial tumor shrinkage observed in patients and thereby contribute to the residual disease state. Clinically, only ~ 1–2% of EGFR mutant LUAD tumors progressing on TKIs exhibit a mesenchymal phenotype [18]. Thus, despite the high frequency with which this program is observed in vitro, TKI-induced mesenchymal reprogrammed cells may exist only transiently during the evolution of acquired drug resistance, although this does not discount the potential importance of this reprogramming response for cancer cell persistence. As discussed below, molecular-based studies on biopsies obtained from cancers early in treatment with oncogene-targeted drugs will be required to assess if and to what degree inhibitor-induced mesenchymal differentiation occurs.

Clinically, emergence of TKI-resistant EGFR mutant LUADs that exhibit small cell lung cancer (SCLC) lineage markers and heralded by RB1 and TP53 loss (Fig. 2) has been observed as another example of a reprogramming response [18, 57–59], although this seems to be a late event in the course of therapy [58]. Notably, these TKI-resistant tumors acquire sensitivity to cytotoxic therapy consistent with SCLC. Studies demonstrate that both tumor subtypes arise from a common EGFR mutant tumor rather than from two distinct cancers, indicating that the tumor has adopted an entirely different cell fate. While no preclinical models have been reported to exhibit this behavior, it seems likely that this will be driven by marked transcriptional reprogramming similar to induction of EMT where switching to the SCLC lineage would relieve cancer cells of their dependence on mutant EGFR.

Overall, these studies indicate that, with sufficient foresight of specific bypass signaling pathways induced in response to TKI-stimulated cellular reprogramming, effective drug combinations could be designed and deployed to bring about greater tumor inhibition. However, in many cases, there appears to be significant diversity and/or redundancy in bypass pathway utilization in different cancer cell lines. For full implementation, the degree of variability in the reprogramming response across an oncogene-defined set of cancers will need to be fully understood. In this regard, a general caveat of the preclinical studies showing TKI-induced reprogramming is that the conclusions are frequently derived from a limited number of cell lines. As further developed below, it will be critical to interrogate the reprogrammed state in primary cancers under treatment to fully appreciate the heterogeneity of response. Moreover, if multiple mechanisms emerge in oncogene-defined cancer subsets, consideration must be given to biomarkers in pretreatment biopsies that may predict a specific reprogramming response.

An alternative approach to combinations of RTK inhibitors with specific bypass pathway inhibitors is to target the driving RTK oncogene in combination with agents that block the reprogramming response at the transcriptional level. As an example, Stuhlmiller et al. demonstrated rapid lapatinib-induced reprogramming in a panel of ERBB2+ breast cancer cell lines [60]. The adaptive responses involved reactivation of ERBB signaling as well as transcriptional upregulation and activation of multiple tyrosine kinases. Their findings showed that inhibition of BET bromodomain chromatin readers with drugs like JQ1 suppressed transcription of many of the lapatinib-induced kinases involved in resistance. Moreover, combining inhibitors of ERBB2 and chromatin readers to prevent kinome reprogramming blocked outgrowth of adapted cancer cells assessed with in vitro assays. Although the combination of lapatinib and chromatin reader inhibitors was not tested in xenograft models, combinations of MAP2K inhibitors and a BRD4 inhibitor, I-BET151, provided improved triple-negative breast cancer xenograft control relative to monotherapies [61]. Clinical grade BRD4 inhibitors [62, 63] have been developed and their single agent activity in cancer patients is presently being tested in clinical trials.

### RTK-induced reprogramming with putative non-tumor cell autonomous functions

**Fig. 3:**
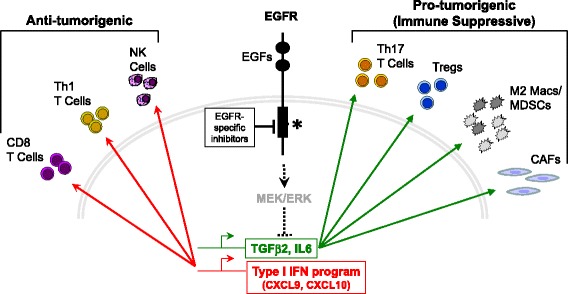
Rapid transcriptional changes in response to oncogenic RTK inhibition may function in a non-cell autonomous manner. Targeted RTK inhibitors stimulate rapid transcriptional induction of TGFβ2, IL6, and a type I IFN program that includes the chemokines, CXCL9 and CXCL10. As shown, these rapidly induced secreted factors are proposed to signal in a paracrine manner to the TME including cancer associated fibroblasts (CAFs) and pro-tumorigenic and anti-tumorigenic cell types of the immune microenvironment. IL6 and TGFβ2 act on the TME to inhibit the activity of CD8+ T cells and increase the recruitment of pro-tumor immune cells including M2-type macrophages and granulocytic myeloid-derived suppressor cells (MDSCs). Additionally, cytokines can increase the activation of CAFs in the TME to increase tumorigenesis. By contrast, the type I IFN response genes, CXCL9 and CXCL10, lead to recruitment of specific T cells and natural killer (NK) cell populations that function in an anti-tumorigenic manner. The balance of these paracrine signals is predicted to contribute to overall tumor growth and survival in the setting of RTK inhibitors, but also to increase vulnerability to distinct immunotherapy strategies

Studies investigating reprogramming responses to RTK inhibitors in cancer cells addicted to specific oncogenic RTKs have tended to largely interpret the results from a cancer cell autonomous viewpoint. It is clear that RTK inhibitor-induced reprogramming induces secretion of myriad factors, some of which may signal in a paracrine fashion to the TME (Fig. 3). In the present era of heightened awareness of the contribution of the TME to cancer cell growth and therapeutic response [64–66], it is important to consider functions of reprogramming that will not be fully appreciated when interpreted from the cell autonomous view. For example, in light of the potent activity of IL6 on many cell types, the aforementioned TKI-induced secretion of this interleukin is likely to initiate paracrine signaling to the TME in addition to autocrine actions on the cancer cells. Caetano et al. [67] demonstrated in KRAS mutant LUAD that IL6 inhibitors reduced autocrine growth and survival signaling on tumor cells, but also markedly altered the lung microenvironment to adopt an anti-tumor phenotype evidenced by reduced pro-tumor immune cells (M2-type macrophages, granulocytic myeloid-derived suppressor cells, and T-regulatory/Th17 cells) and increased anti-tumor Th1 and CD8+ T cells. Similarly, EGFR inhibitor-stimulated production and secretion of TGFβ2 functions as an autocrine growth factor in HNSCC cells [37], but is predicted to exert diverse effects on the TME, including the immune microenvironment. For example, Bedi et al. [68] showed that tumor cell-expressed TGFβ exerts an extrinsic inhibition of the cytotoxic function of immune effectors by suppressing the expression of key molecular effectors including Apo2L/TRAIL, CD95L/FasL, granzyme B, and interferon gamma (IFNγ). Moreover, combinatorial treatment with cetuximab and a TGFβ–blocking antibody resulted in complete tumor regression of HNSCC xenografts. Thus, the literature supports the ability of RTK inhibitor-induced reprogramming through increased secretion of IL6 and TGFβ to enhance immune evasion such that combinations of RTK inhibitors and blockade of IL6 or TGFβ signaling allows participation of the immune response in tumor control.

A well-defined side effect of small molecule and antibody-based inhibitors of EGFR is an acneiform rash [16]. The literature [69–71] reveals that this particular side effect of EGFR inhibitors is related to a suppressive effect of EGFR signaling on the dermal innate immune responses. Pastore and colleagues [69, 72] have shown that EGFR inhibitors induce a type I IFN response in human skin and cultured keratinocytes where the type I IFN response pathway has classically been viewed as an innate cellular response to combat viral infection as well as to communicate with the adaptive immune system through increased CXCL9 and CXCL10 chemokine expression [73, 74]. A microarray analysis of cetuximab-treated A431 cells revealed similar evidence of IFN and STAT1 activation [75]. Pollack et al. [76, 77] demonstrated that EGFR tyrosine kinase inhibitors and cetuximab enhanced induction of MHC class I and II by IFNγ in primary keratinocytes and A431 cells. Notably, increased MHC class I expression was actually independent of IFNγ. Also, skin biopsies from cancer patients exhibited increased epidermal MHC class I protein expression during therapy with an EGFR inhibitor. In a cohort of patients bearing EGFR mutant lung cancers, increased peripheral natural killer cells and INFγ were observed after 4 weeks of gefitinib treatment while circulating IL6 levels were decreased, especially in those patients sensitive to gefitinib [78]. Giles et al. presented evidence of IFN-stimulated gene induction in HNSCC cells adapted for resistance to erlotinib [54]. These published findings are intriguing and suggest that RTK inhibitors activate paracrine communication with the TME through a type I IFN program and potentially influence immune responses through recruitment and activation of the adaptive immune system as well as modulating MHC expression and antigen presentation. In our own studies [56, 79], expression array and RNAseq experiments reveal broad and marked induction of IFN-stimulated genes including CXCL10 in HNSCC cell lines and EGFR mutant lung cancer cell lines treated with EGFR inhibitors as well as EML4-ALK-driven cell lines treated with crizotinib. Combined, the findings support a hypothesis that oncogenic RTKs actively suppress type I IFN pathways, thereby contributing to immune evasion. Thus, testing of rational combinations of RTK inhibitors and immune therapies is supported by these findings.

### Identifying vulnerabilities in persistent tumor cells and development of rational combination therapies

Functional genomics screens have been deployed to provide an unbiased approach to interrogating vulnerabilities in oncogene-driven cancer cells. To identify targets that could be inhibited in combination with EGFR-specific TKIs to yield deeper growth inhibition in EGFR mutant lung cancer cell lines, Casas-Selves et al. [80] used a genome-wide shRNA screen and identified multiple components of the canonical Wnt pathway as contributors to the maintenance of NSCLC cells during EGFR inhibition. Among these, the poly-ADP-ribosylating enzymes tankyrase 1 and 2 that positively regulate canonical Wnt signaling were highlighted. Moreover, inhibition of tankyrase and various other components of the Wnt pathway with shRNAs or small molecules significantly increased the efficacy of EGFR inhibitors both in vitro and in vivo. Subsequently, Scarborough et al. [81] completed a pre-clinical evaluation of a tankyrase inhibitor, AZ1366, in combination with EGFR-specific TKIs in EGFR mutant lung cancer cell lines. In combination with EGFR inhibitors, AZ1366 synergistically suppressed proliferation of multiple lung cancer lines. Also, co-administration of EGFR inhibitor and AZ1366 provided better tumor control and improved survival in mice bearing orthotopic xenografts.

In addition to oncogenic EGFR and distinct RTKs activated through gene-rearrangements, increased expression of the non-mutated RTK, FGFR1, is observed in lung cancers of all histologies [82–86], mesotheliomas [87] and HNSCC [88–91]. To identify additional druggable vulnerabilities to set the stage for more effective combination therapies in FGFR1-dependent cancer, Singleton et al. [92] deployed kinome-targeting shRNA libraries to screen for protein kinase pathways that would significantly increase sensitivity to FGFR-specific TKIs. The screens identified MTOR as a high-ranking synthetic lethal hit in the setting of FGFR-specific TKIs in FGFR1-driven lung cancer and HNSCC cell lines. Importantly, in vivo studies demonstrated increased anti-tumor activity of FGFR TKIs in combination with MTOR inhibitors using xenograft models.

A study by Harbinski et al. [93] used a cDNA library encoding secreted proteins to systematically assess the potential of secreted proteins including diverse growth factors to induce resistance to kinase inhibitors in cancer cell lines highly addicted to MET, FGFR2 or FGFR3. The results revealed ligand-mediated activation of alternative RTK expressed on TKI-naïve cancer cells that functioned as bypass pathways to MET and FGFR-specific TKIs. The implication of this study is that relevant growth and survival signaling can arise through ligand-mediated paracrine communication between cancer cells and the TME. Moreover, these signal pathways will not be identified with in vitro assays or screens. As a potentially relevant example, published studies reveal exquisite in vitro sensitivity of FGFR1-dependent cancer cell lines to FGFR-specific TKIs, although their in vivo sensitivity to these drugs was rather modest [87, 92]. In this regard, the reduced in vivo sensitivity to FGFR-specific TKIs is consistent with results of clinical trials in FGFR positive solid tumors where only partial responses have been observed in ~ 10% of patients [94–97]. Our group is presently exploring the hypothesis that FGFR-dependent cancers receive significant paracrine input from the TME that diminishes the degree of dependency on over-expressed or oncogenically mutated FGFR pathways. Our approach involves functional RNAi screens, but in the in vivo setting using orthotopic xenograft models with the goal of identifying receptor pathways that yield synthetic lethal phenotypes in FGFR-specific TKI-treated xenograft tumors.

### Perspectives and priorities

#### Defining TKI-induced reprogramming in primary specimens from tumors under treatment with oncogene targeted therapies

Numerous studies have examined mechanisms of acquired resistance presenting at tumor progression which likely reflect outgrowth of dominant evolutionarily-selected clones. By contrast, much less is known about signaling and transcriptional mechanisms in the setting of early residual disease following oncogene inhibition, in part because patient biopsies are rarely performed early during response to therapy [23]. Obviously, the molecular evaluation of the residual disease state in primary tumor specimens early during oncogene-targeted therapy would provide a multilayered view of the cancer-TME “ecosystem” that ultimately drives resistance in patients. Also, in keeping with the topics discussed above, the resistance-conferring features of both tumor cell autonomous and non-autonomous signaling could be explored. In this regard, Song et al. [98, 99] recently published an extensive analysis of the transcriptomes of primary melanoma regressing on MAPK inhibitor therapy. Residual tumors displayed evidence of mesenchymal, angiogenic, and IFN pathway reprogramming as well as growth and survival dependence on multiple RTKs and PD-L2, an immune checkpoint protein. This comprehensive inspection of residual melanoma early in therapy illuminates multiple putative vulnerabilities that can be reverse translated to laboratory-based studies using murine models of melanoma.

Similar “window of opportunity” trials are being launched to study remnant lung tumor samples after oncogene targeted therapies. McCoach and colleagues [100] are implementing a clinical trial (NCT03088930) in which patients with early stage lung cancers bearing activating mutations in ALK, ROS1 or MET exon 14 are treated with neoadjuvant crizotinib. After 6 weeks of therapy, definitive surgical resection is performed, thereby providing patient benefit, but also primary tumor tissue for multiple molecular-based analyses. Also, a clinical trial entitled “*Early Rebiopsy to Identify Biomarkers of Tumor Cell Survival Following EGFR TKI Therapy* (NCT03042221)” will molecularly analyze paired baseline biopsy specimens from advanced stage EGFR mutant lung cancers and biopsies obtained following 2 weeks of EGFR TKI-targeted treatment with the intention to identify early adaptive mechanisms of cell survival in the setting of oncogene-targeted therapy. Preliminary RNAseq analysis of two sets of paired biopsies reveals evidence of both tumor cell autonomous and non-autonomous transcriptional responses [56]. Analysis of a larger set of samples is predicted to shed considerable light on the heterogeneity of the reprogramming response in residual EGFR mutant lung cancer. A search of clinicaltrials.gov indicates other similar neoadjuvant trials based on oncogene-targeted agents have either been completed or are open and active (*Surgery for Early Lung Cancer With Preoperative Erlotinib (Tarceva): A Clinical Phase II Trial (SELECT)*, NCT00462995 (completed, no data posted); *Study of TARCEVA (Erlotinib) as Adjuvant Treatment for Locally Advanced Head and Neck Squamous Cell Carcinoma*, NCT01515137 (completed, no data posted)). These clinical protocols with specific intention to collect samples of tumors early during targeted therapy are anticipated to provide a clearer molecular understanding of residual cancer that will prompt informative laboratory studies.

#### Immune competent murine models of oncogene-driven solid cancers for reverse translational studies

The aforementioned clinical protocols will provide rich insight into the biology of residual disease and potentially drive reverse translational research [101] to explore molecular and cellular mechanisms. Success in this endeavor demands rigorous models of oncogene-driven cancers that permit testing of both cancer cell autonomous and non-autonomous signaling mediating tumor cell persistence. Genetically engineered mouse models of oncogene-driven cancers appear to generate tumors that lack the mutation burden typified by their human equivalents and therefore, may not fully reflect the conversation between cancer cells and the immune microenvironment [102]. Patient-derived xenograft (PDX) models require humanized murine hosts which continue to undergo optimization [103]. Moreover, while PDXs are presumed to more faithfully represent primary cancers compared to tumor-derived cell line models, a recent report reveals that the molecular features of PDXs diverge substantially from the parental tumors during passage [104]. As alternatives to these approaches, we favor established murine cancer cell lines that bear relevant mutation burdens and can be implanted into immune competent hosts to enable full communication with the TME, including the immune microenvironment [105–107]. Advantages of implantable murine cancer cell line models are the ability to manipulate them with molecular biological techniques and complete in vivo testing in a fully immune competent host. A disadvantage is the paucity of oncogene-driven murine cancer cell lines that serve as models for the human disease. However, the characterization of the mutation landscape of different solid tumors coupled with the power of CRISPR/Cas9 techniques to engineer specific oncogenic mutations in mice [108] provides a path forward for development of additional murine cancer cell lines that can model relevant subsets of human oncogene-driven cancers. Murine cancer cell lines established from EML4-ALK-positive tumors initiated with CRISPR/Cas9 techniques reveals that they retain the predicted oncogene-addicted state, bear relevant mutation burden and exhibit responses to immunotherapy consistent with the human disease [105, 108]. If successful, these models may unveil RTK inhibitor responses in a fully immune competent TME that will permit rigorous evaluation of rationally-based drug combinations to greatly reduce or eliminate the residual disease observed with monotherapy strategies.

## Conclusions

The clinical experience with TKI monotherapy in cancers bearing oncogenic RTKs highlights the importance of defining next-generation strategies that will reduce or eliminate acquired resistance. While serial deployment of 2nd and 3rd generation TKIs may increase the duration of response, resistance still eventually emerges due to residual disease. Based on the studies reviewed in this article, approaches whereby novel and rational “upfront” combinations of TKIs and drugs targeting signal pathways that prevent rapid tumor cell reprogramming must be identified and prioritized for preclinical testing. Evidence that blocking oncogenic pathways within cancer cells unleashes paracrine signaling between cancer cells and the TME, including the innate and adaptive immune systems, supports the continued development of transplantable murine cancer cell lines that faithfully represent oncogene-driven human cancers for subsequent analysis of the impact of TME-cancer cell crosstalk on TKI efficacy in fully immune competent hosts. This latter approach will permit rational evaluation of combinations of TKIs with drugs targeting TME pathways as well as approved and emerging immunotherapeutics. Finally, these studies must be accompanied by deep evaluation of molecular responses in primary human tumors early during TKI treatment to determine the kinetics of the processes as well as the degree of variation across tumor subtypes. Combined, these strategies may yield novel combination therapies that maximize the initial tumor shrinkage response, thereby leading to stable disease or perhaps cures.

## Abbreviations

ALK: Anaplastic lymphoma kinase
BET: Bromodomain and extra-terminal (BET) family proteins
BRD4: Bromodomain containing 4
CDH1: Cadherin 1
EGFR: Epidermal growth factor receptor
EML4: Echinoderm microtubule associated protein like 4
EMT: Epithelial to mesenchymal transition
ERBB2: erb-b2 receptor tyrosine kinase 2
ERK: Extracellular signal regulated kinase
ESRP1: Epithelial splicing regulatory protein 1
FGFR: Fibroblast growth factor receptor
HNSCC: Head and neck squamous cell carcinoma
IFN: Interferon
IL6: Interleukin 6
JAK: Janus kinase
LUAD: Lung adenocarcinoma
MAP2K: Mitogen-activated protein kinase kinase
MAPK: Mitogen-activated protein kinase
MEK: MAP/ERK kinase
MET: MET proto-oncogene receptor tyrosine kinase
MTOR: Mammalian target of rapamycin
NTRK1: Neurotrophic receptor tyrosine kinase 1
PDGFR: Platelet-derived growth factor receptor
RET: RET proto-oncogene receptor tyrosine kinase
ROS1: ROS proto-oncogene 1 receptor tyrosine kinase
RTK: Receptor tyrosine kinase
SCLC: Small cell lung cancer
STAT: Signal transducer and activator of transcription
TGFβ: Transforming growth factor beta
TGFβR: Transforming growth factor beta receptor
TKI: Tyrosine kinase inhibitor
TME: Tumor microenvironment
TORC: Target of rapamycin complex

## References

[1] Camidge DR, Bang YJ, Kwak EL, Iafrate AJ, Varella-Garcia M, Fox SB, Riely GJ, Solomon B, Ou SH, Kim DW (2012). Activity and safety of crizotinib in patients with ALK-positive non-small-cell lung cancer: updated results from a phase 1 study. Lancet Oncol.

[2] Camidge DR, Doebele RC (2012). Treating ALK-positive lung cancer--early successes and future challenges. Nat Rev Clin Oncol.

[3] Davies KD, Le AT, Theodoro MF, Skokan MC, Aisner DL, Berge EM, Terracciano LM, Cappuzzo F, Incarbone M, Roncalli M (2012). Identifying and targeting ROS1 gene fusions in non-small cell lung cancer. Clin Cancer Res.

[4] Mok TS, Wu YL, Thongprasert S, Yang CH, Chu DT, Saijo N, Sunpaweravong P, Han B, Margono B, Ichinose Y (2009). Gefitinib or carboplatin-paclitaxel in pulmonary adenocarcinoma. N Engl J Med.

[5] Shaw AT, Kim DW, Mehra R, Tan DS, Felip E, Chow LQ, Camidge DR, Vansteenkiste J, Sharma S, De Pas T (2014). Ceritinib in ALK-rearranged non-small-cell lung cancer. N Engl J Med.

[6] Shaw AT, Ou SH, Bang YJ, Camidge DR, Solomon BJ, Salgia R, Riely GJ, Varella-Garcia M, Shapiro GI, Costa DB (2014). Crizotinib in ROS1-rearranged non-small-cell lung cancer. N Engl J Med.

[7] Solomon BJ, Mok T, Kim DW, Wu YL, Nakagawa K, Mekhail T, Felip E, Cappuzzo F, Paolini J, Usari T (2014). First-line crizotinib versus chemotherapy in ALK-positive lung cancer. N Engl J Med.

[8] Yang JC, Wu YL, Schuler M, Sebastian M, Popat S, Yamamoto N, Zhou C, Hu CP, O'Byrne K, Feng J (2015). Afatinib versus cisplatin-based chemotherapy for EGFR mutation-positive lung adenocarcinoma (LUX-Lung 3 and LUX-Lung 6): analysis of overall survival data from two randomised, phase 3 trials. Lancet Oncol.

[9] Drilon A, Siena S, Ou SI, Patel M, Ahn MJ, Lee J, Bauer TM, Farago AF, Wheler JJ, Liu SV (2017). Safety and Antitumor Activity of the Multitargeted Pan-TRK, ROS1, and ALK Inhibitor Entrectinib: Combined Results from Two Phase I Trials (ALKA-372-001 and STARTRK-1). Cancer Discov.

[10] Frampton GM, Ali SM, Rosenzweig M, Chmielecki J, Lu X, Bauer TM, Akimov M, Bufill JA, Lee C, Jentz D (2015). Activation of MET via diverse exon 14 splicing alterations occurs in multiple tumor types and confers clinical sensitivity to MET inhibitors. Cancer Discov.

[11] Planchard D, Kim TM, Mazieres J, Quoix E, Riely G, Barlesi F, Souquet PJ, Smit EF, Groen HJ, Kelly RJ (2016). Dabrafenib in patients with BRAF(V600E)-positive advanced non-small-cell lung cancer: a single-arm, multicentre, open-label, phase 2 trial. Lancet Oncol.

[12] Vaishnavi A, Capelletti M, Le AT, Kako S, Butaney M, Ercan D, Mahale S, Davies KD, Aisner DL, Pilling AB (2013). Oncogenic and drug-sensitive NTRK1 rearrangements in lung cancer. Nat Med.

[13] Vermorken JB, Trigo J, Hitt R, Koralewski P, Diaz-Rubio E, Rolland F, Knecht R, Amellal N, Schueler A, Baselga J (2007). Open-label, uncontrolled, multicenter phase II study to evaluate the efficacy and toxicity of cetuximab as a single agent in patients with recurrent and/or metastatic squamous cell carcinoma of the head and neck who failed to respond to platinum-based therapy. J Clin Oncol.

[14] Vermorken JB, Mesia R, Rivera F, Remenar E, Kawecki A, Rottey S, Erfan J, Zabolotnyy D, Kienzer HR, Cupissol D (2008). Platinum-based chemotherapy plus cetuximab in head and neck cancer. N Engl J Med.

[15] Bonner JA, Harari PM, Giralt J, Azarnia N, Shin DM, Cohen RB, Jones CU, Sur R, Raben D, Jassem J (2006). Radiotherapy plus cetuximab for squamous-cell carcinoma of the head and neck. N Engl J Med.

[16] Bonner JA, Harari PM, Giralt J, Cohen RB, Jones CU, Sur RK, Raben D, Baselga J, Spencer SA, Zhu J (2010). Radiotherapy plus cetuximab for locoregionally advanced head and neck cancer: 5-year survival data from a phase 3 randomised trial, and relation between cetuximab-induced rash and survival. Lancet Oncol.

[17] Gingras I, Gebhart G, de Azambuja E, Piccart-Gebhart M (2017). HER2-positive breast cancer is lost in translation: time for patient-centered research. Nat Rev Clin Oncol.

[18] Camidge DR, Pao W, Sequist LV (2014). Acquired resistance to TKIs in solid tumours: learning from lung cancer. Nat Rev Clin Oncol.

[19] Lovly CM, Shaw AT (2014). Molecular pathways: resistance to kinase inhibitors and implications for therapeutic strategies. Clin Cancer Res.

[20] Yu HA, Arcila ME, Rekhtman N, Sima CS, Zakowski MF, Pao W, Kris MG, Miller VA, Ladanyi M, Riely GJ (2013). Analysis of tumor specimens at the time of acquired resistance to EGFR-TKI therapy in 155 patients with EGFR-mutant lung cancers. Clin Cancer Res.

[21] Janne PA, Yang JC, Kim DW, Planchard D, Ohe Y, Ramalingam SS, Ahn MJ, Kim SW, Su WC, Horn L (2015). AZD9291 in EGFR inhibitor-resistant non-small-cell lung cancer. N Engl J Med.

[22] Glickman MS, Sawyers CL (2012). Converting cancer therapies into cures: lessons from infectious diseases. Cell.

[23] Bivona TG, Doebele RC (2016). A framework for understanding and targeting residual disease in oncogene-driven solid cancers. Nat Med.

[24] Sharma SV, Lee DY, Li B, Quinlan MP, Takahashi F, Maheswaran S, McDermott U, Azizian N, Zou L, Fischbach MA (2010). A chromatin-mediated reversible drug-tolerant state in cancer cell subpopulations. Cell.

[25] Jamal-Hanjani M, Wilson GA, McGranahan N, Birkbak NJ, Watkins TBK, Veeriah S, Shafi S, Johnson DH, Mitter R, Rosenthal R (2017). Tracking the Evolution of Non-Small-Cell Lung Cancer. N Engl J Med.

[26] McCoach CE, Blumenthal GM, Zhang L, Myers A, Tang S, Sridhara R, Keegan P, Pazdur R, Doebele RC, Kazandjian D (2017). Exploratory analysis of the association of depth of response and survival in patients with metastatic non-small-cell lung cancer treated with a targeted therapy or immunotherapy. Ann Oncol.

[27] Manning G, Whyte DB, Martinez R, Hunter T, Sudarsanam S (2002). The protein kinase complement of the human genome. Science.

[28] Graves LM, Duncan JS, Whittle MC, Johnson GL (2013). The dynamic nature of the kinome. Biochem J.

[29] Sun C, Bernards R (2014). Feedback and redundancy in receptor tyrosine kinase signaling: relevance to cancer therapies. Trends Biochem Sci.

[30] Housden BE, Perrimon N (2014). Spatial and temporal organization of signaling pathways. Trends Biochem Sci.

[31] Xu AM, Huang PH (2010). Receptor tyrosine kinase coactivation networks in cancer. Cancer Res.

[32] Ware KE, Marshall ME, Heasley LR, Marek L, Hinz TK, Hercule P, Helfrich BA, Doebele RC, Heasley LE (2010). Rapidly Acquired Resistance to EGFR Tyrosine Kinase Inhibitors in NSCLC Cell Lines through De-Repression of FGFR2 and FGFR3 Expression. PLoS One.

[33] Sharifnia T, Rusu V, Piccioni F, Bagul M, Imielinski M, Cherniack AD, Pedamallu CS, Wong B, Wilson FH, Garraway LA (2014). Genetic modifiers of EGFR dependence in non-small cell lung cancer. Proc Natl Acad Sci U S A.

[34] Akhavan D, Pourzia AL, Nourian AA, Williams KJ, Nathanson D, Babic I, Villa GR, Tanaka K, Nael A, Yang H (2013). De-repression of PDGFRbeta transcription promotes acquired resistance to EGFR tyrosine kinase inhibitors in glioblastoma patients. Cancer Discov.

[35] Lee HJ, Zhuang G, Cao Y, Du P, Kim HJ, Settleman J (2014). Drug resistance via feedback activation of Stat3 in oncogene-addicted cancer cells. Cancer Cell.

[36] Blakely CM, Pazarentzos E, Olivas V, Asthana S, Yan JJ, Tan I, Hrustanovic G, Chan E, Lin L, Neel DS (2015). NF-kappaB-activating complex engaged in response to EGFR oncogene inhibition drives tumor cell survival and residual disease in lung cancer. Cell Rep.

[37] Kleczko EK, Kim J, Keysar SB, Heasley LR, Eagles JR, Simon M, Marshall ME, Singleton KR, Jimeno A, Tan AC, Heasley LE (2015). An Inducible TGF-beta2-TGFbetaR Pathway Modulates the Sensitivity of HNSCC Cells to Tyrosine Kinase Inhibitors Targeting Dominant Receptor Tyrosine Kinases. PLoS One.

[38] Zhang G, Scarborough H, Kim J, Rozhok AI, Chen YA, Zhang X, Song L, Bai Y, Fang B, Liu RZ (2016). Coupling an EML4-ALK-centric interactome with RNA interference identifies sensitizers to ALK inhibitors. Sci Signal.

[39] Vaishnavi A, Schubert L, Rix U, Marek LA, Le AT, Keysar SB, Glogowska MJ, Smith MA, Kako S, Sumi NJ (2017). EGFR Mediates Responses to Small-Molecule Drugs Targeting Oncogenic Fusion Kinases. Cancer Res.

[40] Singleton KR, Kim J, Hinz TK, Marek LA, Casas-Selves M, Hatheway C, Tan AC, DeGregori J, Heasley LE (2013). A receptor tyrosine kinase network composed of fibroblast growth factor receptors, epidermal growth factor receptor, v-erb-b2 erythroblastic leukemia viral oncogene homolog 2, and hepatocyte growth factor receptor drives growth and survival of head and neck squamous carcinoma cell lines. Mol Pharmacol.

[41] Tan AC, Vyse S, Huang PH (2017). Exploiting receptor tyrosine kinase co-activation for cancer therapy. Drug Discov Today.

[42] Nieto MA, Huang RY, Jackson RA, Thiery JP (2016). Emt: 2016. Cell.

[43] Chung JH, Rho JK, Xu X, Lee JS, Yoon HI, Lee CT, Choi YJ, Kim HR, Kim CH, Lee JC (2011). Clinical and molecular evidences of epithelial to mesenchymal transition in acquired resistance to EGFR-TKIs. Lung Cancer.

[44] Suda K, Tomizawa K, Fujii M, Murakami H, Osada H, Maehara Y, Yatabe Y, Sekido Y, Mitsudomi T (2011). Epithelial to mesenchymal transition in an epidermal growth factor receptor-mutant lung cancer cell line with acquired resistance to erlotinib. J Thorac Oncol.

[45] Ware KE, Hinz TK, Kleczko E, Singleton KR, Marek LA, Helfrich BA, Cummings CT, Graham DK, Astling D, Tan AC, Heasley LE (2013). A mechanism of resistance to gefitinib mediated by cellular reprogramming and the acquisition of an FGF2-FGFR1 autocrine growth loop. Oncogene.

[46] Yoshida T, Song L, Bai Y, Kinose F, Li J, Ohaegbulam KC, Munoz-Antonia T, Qu X, Eschrich S, Uramoto H (2016). ZEB1 Mediates Acquired Resistance to the Epidermal Growth Factor Receptor-Tyrosine Kinase Inhibitors in Non-Small Cell Lung Cancer. PLoS One.

[47] Brand TM, Iida M, Wheeler DL (2011). Molecular mechanisms of resistance to the EGFR monoclonal antibody cetuximab. Cancer Biol Ther.

[48] Maseki S, Ijichi K, Tanaka H, Fujii M, Hasegawa Y, Ogawa T, Murakami S, Kondo E, Nakanishi H (2012). Acquisition of EMT phenotype in the gefitinib-resistant cells of a head and neck squamous cell carcinoma cell line through Akt/GSK-3beta/snail signalling pathway. Br J Cancer.

[49] Thomson S, Petti F, Sujka-Kwok I, Epstein D, Haley JD (2008). Kinase switching in mesenchymal-like non-small cell lung cancer lines contributes to EGFR inhibitor resistance through pathway redundancy. Clin Exp Metastasis.

[50] Thomson S, Petti F, Sujka-Kwok I, Mercado P, Bean J, Monaghan M, Seymour SL, Argast GM, Epstein DM, Haley JD (2011). A systems view of epithelial-mesenchymal transition signaling states. Clin Exp Metastasis.

[51] Postel-Vinay S, Ashworth A (2012). AXL and acquired resistance to EGFR inhibitors. Nat Genet.

[52] Zhang Z, Lee JC, Lin L, Olivas V, Au V, LaFramboise T, Abdel-Rahman M, Wang X, Levine AD, Rho JK (2012). Activation of the AXL kinase causes resistance to EGFR-targeted therapy in lung cancer. Nat Genet.

[53] Brand TM, Iida M, Stein AP, Corrigan KL, Braverman CM, Luthar N, Toulany M, Gill PS, Salgia R, Kimple RJ, Wheeler DL (2014). AXL mediates resistance to cetuximab therapy. Cancer Res.

[54] Giles KM, Kalinowski FC, Candy PA, Epis MR, Zhang PM, Redfern AD, Stuart LM, Goodall GJ, Leedman PJ (2013). Axl mediates acquired resistance of head and neck cancer cells to the epidermal growth factor receptor inhibitor erlotinib. Mol Cancer Ther.

[55] Debruyne DN, Bhatnagar N, Sharma B, Luther W, Moore NF, Cheung NK, Gray NS, George RE (2016). ALK inhibitor resistance in ALK(F1174L)-driven neuroblastoma is associated with AXL activation and induction of EMT. Oncogene.

[56] Gurule NJ, McCoach C, Hinz TK, Marek L, Ryall K, Korpela S, Sisler D, Tan AC, Doebele RC, Heasley LE (2018). Oncogene-targeted agents induce an interferon response in EGFR and EML4-ALK driven lung cancer.

[57] Sequist LV, Waltman BA, Dias-Santagata D, Digumarthy S, Turke AB, Fidias P, Bergethon K, Shaw AT, Gettinger S, Cosper AK (2011). Genotypic and histological evolution of lung cancers acquiring resistance to EGFR inhibitors. Sci Transl Med.

[58] Roca E, Gurizzan C, Amoroso V, Vermi W, Ferrari V, Berruti A (2017). Outcome of patients with lung adenocarcinoma with transformation to small-cell lung cancer following tyrosine kinase inhibitors treatment: A systematic review and pooled analysis. Cancer Treat Rev.

[59] Lee JK, Lee J, Kim S, Kim S, Youk J, Park S, An Y, Keam B, Kim DW, Heo DS (2017). Clonal History and Genetic Predictors of Transformation Into Small-Cell Carcinomas From Lung Adenocarcinomas. J Clin Oncol.

[60] Stuhlmiller TJ, Miller SM, Zawistowski JS, Nakamura K, Beltran AS, Duncan JS, Angus SP, Collins KA, Granger DA, Reuther RA (2015). Inhibition of Lapatinib-Induced Kinome Reprogramming in ERBB2-Positive Breast Cancer by Targeting BET Family Bromodomains. Cell Rep.

[61] Zawistowski JS, Bevill SM, Goulet DR, Stuhlmiller TJ, Beltran AS, Olivares-Quintero JF, Singh D, Sciaky N, Parker JS, Rashid NU (2017). Enhancer Remodeling during Adaptive Bypass to MEK Inhibition Is Attenuated by Pharmacologic Targeting of the P-TEFb Complex. Cancer Discov.

[62] Rhyasen GW, Hattersley MM, Yao Y, Dulak A, Wang W, Petteruti P, Dale IL, Boiko S, Cheung T, Zhang J (2016). AZD5153: A Novel Bivalent BET Bromodomain Inhibitor Highly Active against Hematologic Malignancies. Mol Cancer Ther.

[63] Mirguet O, Gosmini R, Toum J, Clement CA, Barnathan M, Brusq JM, Mordaunt JE, Grimes RM, Crowe M, Pineau O (2013). Discovery of epigenetic regulator I-BET762: lead optimization to afford a clinical candidate inhibitor of the BET bromodomains. J Med Chem.

[64] Bizzarri M, Cucina A (2014). Tumor and the microenvironment: a chance to reframe the paradigm of carcinogenesis?. Biomed Res Int.

[65] Pitt JM, Marabelle A, Eggermont A, Soria JC, Kroemer G, Zitvogel L (2016). Targeting the tumor microenvironment: removing obstruction to anticancer immune responses and immunotherapy. Ann Oncol.

[66] Singh SR, Rameshwar P, Siegel P (2016). Targeting tumor microenvironment in cancer therapy. Cancer Lett.

[67] Caetano MS, Zhang H, Cumpian AM, Gong L, Unver N, Ostrin EJ, Daliri S, Chang SH, Ochoa CE, Hanash S (2016). IL6 Blockade Reprograms the Lung Tumor Microenvironment to Limit the Development and Progression of K-ras-Mutant Lung Cancer. Cancer Res.

[68] Bedi A, Chang X, Noonan K, Pham V, Bedi R, Fertig EJ, Considine M, Califano JA, Borrello I, Chung CH (2012). Inhibition of TGF-beta enhances the in vivo antitumor efficacy of EGF receptor-targeted therapy. Mol Cancer Ther.

[69] Pastore S, Lulli D, Girolomoni G (2014). Epidermal growth factor receptor signalling in keratinocyte biology: implications for skin toxicity of tyrosine kinase inhibitors. Arch Toxicol.

[70] Lichtenberger BM, Gerber PA, Holcmann M, Buhren BA, Amberg N, Smolle V, Schrumpf H, Boelke E, Ansari P, Mackenzie C (2013). Epidermal EGFR controls cutaneous host defense and prevents inflammation. Sci Transl Med.

[71] Mascia F, Lam G, Keith C, Garber C, Steinberg SM, Kohn E, Yuspa SH (2013). Genetic ablation of epidermal EGFR reveals the dynamic origin of adverse effects of anti-EGFR therapy. Sci Transl Med.

[72] Lulli D, Carbone ML, Pastore S (2016). Epidermal growth factor receptor inhibitors trigger a type I interferon response in human skin. Oncotarget.

[73] Ivashkiv LB, Donlin LT (2014). Regulation of type I interferon responses. Nat Rev Immunol.

[74] Spranger S, Gajewski TF (2016). Tumor-intrinsic oncogene pathways mediating immune avoidance. Oncoimmunology.

[75] Oliveras-Ferraros C, Vazquez-Martin A, Queralt B, Adrados M, Ortiz R, Cufi S, Hernandez-Yague X, Guardeno R, Baez L, Martin-Castillo B (2011). Interferon/STAT1 and neuregulin signaling pathways are exploratory biomarkers of cetuximab (Erbitux(R)) efficacy in KRAS wild-type squamous carcinomas: a pathway-based analysis of whole human-genome microarray data from cetuximab-adapted tumor cell-line models. Int J Oncol.

[76] Pollack BP (2012). EGFR inhibitors, MHC expression and immune responses : Can EGFR inhibitors be used as immune response modifiers?. Oncoimmunology.

[77] Pollack BP, Sapkota B, Cartee TV (2011). Epidermal growth factor receptor inhibition augments the expression of MHC class I and II genes. Clin Cancer Res.

[78] Sheng J, Fang W, Liu X, Xing S, Zhan J, Ma Y, Huang Y, Zhou N, Zhao H, Zhang L (2017). Impact of gefitinib in early stage treatment on circulating cytokines and lymphocytes for patients with advanced non-small cell lung cancer. Onco Targets Ther.

[79] Korpela S, Hinz TK, Nemenoff RA, Karam SD, Oweida A, Heasley LE (2018). EGFR Activation Contributes to Immune Evasion in HNSCC.

[80] Casas-Selves M, Kim J, Zhang Z, Helfrich BA, Gao D, Porter CC, Scarborough HA, Bunn PA, Chan DC, Tan AC, DeGregori J (2012). Tankyrase and the canonical Wnt pathway protect lung cancer cells from EGFR inhibition. Cancer Res.

[81] Scarborough HA, Helfrich BA, Casas-Selves M, Schuller AG, Grosskurth SE, Kim J, Tan AC, Chan DC, Zhang Z, Zaberezhnyy V (2017). AZ1366: An Inhibitor of Tankyrase and the Canonical Wnt Pathway that Limits the Persistence of Non-Small Cell Lung Cancer Cells Following EGFR Inhibition. Clin Cancer Res.

[82] Goke F, Franzen A, Menon R, Goltz D, Kirsten R, Boehm D, Vogel W, Goke A, Scheble V, Ellinger J (2012). Rationale for treatment of metastatic squamous cell carcinoma of the lung using fibroblast growth factor receptor inhibitors. Chest.

[83] Marek L, Ware KE, Fritzsche A, Hercule P, Helton WR, Smith JE, McDermott LA, Coldren CD, Nemenoff RA, Merrick DT (2009). Fibroblast growth factor (FGF) and FGF receptor-mediated autocrine signaling in non-small-cell lung cancer cells. Mol Pharmacol.

[84] Wynes MW, Hinz TK, Gao D, Martini M, Marek LA, Ware KE, Edwards MG, Bohm D, Perner S, Helfrich BA (2014). FGFR1 mRNA and protein expression, not gene copy number, predict FGFR TKI sensitivity across all lung cancer histologies. Clin Cancer Res.

[85] Dutt A, Ramos AH, Hammerman PS, Mermel C, Cho J, Sharifnia T, Chande A, Tanaka KE, Stransky N, Greulich H (2011). Inhibitor-sensitive FGFR1 amplification in human non-small cell lung cancer. PLoS One.

[86] Weiss J, Sos ML, Seidel D, Peifer M, Zander T, Heuckmann JM, Ullrich RT, Menon R, Maier S, Soltermann A (2010). Frequent and focal FGFR1 amplification associates with therapeutically tractable FGFR1 dependency in squamous cell lung cancer. Sci Transl Med.

[87] Marek LA, Hinz TK, von Massenhausen A, Olszewski KA, Kleczko EK, Boehm D, Weiser-Evans MC, Nemenoff RA, Hoffmann H, Warth A (2014). Nonamplified FGFR1 is a growth driver in malignant pleural mesothelioma. Mol Cancer Res.

[88] Goke F, Bode M, Franzen A, Kirsten R, Goltz D, Goke A, Sharma R, Boehm D, Vogel W, Wagner P (2013). Fibroblast growth factor receptor 1 amplification is a common event in squamous cell carcinoma of the head and neck. Mod Pathol.

[89] Goke F, Franzen A, Hinz TK, Marek LA, Yoon P, Sharma R, Bode M, von Maessenhausen A, Lankat-Buttgereit B, Goke A (2015). FGFR1 Expression Levels Predict BGJ398 Sensitivity of FGFR1-Dependent Head and Neck Squamous Cell Cancers. Clin Cancer Res.

[90] Marshall ME, Hinz TK, Kono SA, Singleton KR, Bichon B, Ware KE, Marek L, Frederick BA, Raben D, Heasley LE (2011). Fibroblast growth factor receptors are components of autocrine signaling networks in head and neck squamous cell carcinoma cells. Clin Cancer Res.

[91] Freier K, Schwaenen C, Sticht C, Flechtenmacher C, Muhling J, Hofele C, Radlwimmer B, Lichter P, Joos S (2007). Recurrent FGFR1 amplification and high FGFR1 protein expression in oral squamous cell carcinoma (OSCC). Oral Oncol.

[92] Singleton KR, Hinz TK, Kleczko EK, Marek LA, Kwak J, Harp T, Kim J, Tan AC, Heasley LE (2015). Kinome RNAi Screens Reveal Synergistic Targeting of MTOR and FGFR1 Pathways for Treatment of Lung Cancer and HNSCC. Cancer Res.

[93] Harbinski F, Craig VJ, Sanghavi S, Jeffery D, Liu L, Sheppard KA, Wagner S, Stamm C, Buness A, Chatenay-Rivauday C (2012). Rescue screens with secreted proteins reveal compensatory potential of receptor tyrosine kinases in driving cancer growth. Cancer Discov.

[94] Javle M, Lowery M, Shroff RT, Weiss KH, Springfeld C, Borad MJ, Ramanathan RK, Goyal L, Sadeghi S, Macarulla T, et al. Phase II Study of BGJ398 in Patients With FGFR-Altered Advanced Cholangiocarcinoma. J Clin Oncol. 2018;36:276–82. 10.1200/JCO.2017.75.5009 PMC6075847 29182496

[95] Nogova L, Sequist LV, Perez Garcia JM, Andre F, Delord JP, Hidalgo M, Schellens JH, Cassier PA, Camidge DR, Schuler M (2017). Evaluation of BGJ398, a Fibroblast Growth Factor Receptor 1-3 Kinase Inhibitor, in Patients With Advanced Solid Tumors Harboring Genetic Alterations in Fibroblast Growth Factor Receptors: Results of a Global Phase I, Dose-Escalation and Dose-Expansion Study. J Clin Oncol.

[96] Paik PK, Shen R, Berger MF, Ferry D, Soria JC, Mathewson A, Rooney C, Smith NR, Cullberg M, Kilgour E (2017). A Phase Ib Open-Label Multicenter Study of AZD4547 in Patients with Advanced Squamous Cell Lung Cancers. Clin Cancer Res.

[97] Van Cutsem E, Bang YJ, Mansoor W, Petty RD, Chao Y, Cunningham D, Ferry DR, Smith NR, Frewer P, Ratnayake J (2017). A randomized, open-label study of the efficacy and safety of AZD4547 monotherapy versus paclitaxel for the treatment of advanced gastric adenocarcinoma with FGFR2 polysomy or gene amplification. Ann Oncol.

[98] Song C, Piva M, Sun L, Hong A, Moriceau G, Kong X, Zhang H, Lomeli S, Qian J, Yu CC (2017). Recurrent Tumor Cell-Intrinsic and -Extrinsic Alterations during MAPKi-Induced Melanoma Regression and Early Adaptation. Cancer Discov.

[99] Haq R (2017). Trapping Cancers as They Adapt to Survive. Cancer Discov.

[100] McCoach CE, Bivona TG, Blakely CM, Doebele RC (2016). Neoadjuvant Oncogene-Targeted Therapy in Early Stage Non-Small-Cell Lung Cancer as a Strategy to Improve Clinical Outcome and Identify Early Mechanisms of Resistance. Clin Lung Cancer.

[101] Ledford H (2008). Translational research: the full cycle. Nature.

[102] McFadden DG, Politi K, Bhutkar A, Chen FK, Song X, Pirun M, Santiago PM, Kim-Kiselak C, Platt JT, Lee E (2016). Mutational landscape of EGFR-, MYC-, and Kras-driven genetically engineered mouse models of lung adenocarcinoma. Proc Natl Acad Sci U S A.

[103] Herndler-Brandstetter D, Shan L, Yao Y, Stecher C, Plajer V, Lietzenmayer M, Strowig T, de Zoete MR, Palm NW, Chen J (2017). Humanized mouse model supports development, function, and tissue residency of human natural killer cells. Proc Natl Acad Sci U S A.

[104] Ben-David U, Ha G, Tseng YY, Greenwald NF, Oh C, Shih J, McFarland JM, Wong B, Boehm JS, Beroukhim R, Golub TR (2017). Patient-derived xenografts undergo mouse-specific tumor evolution. Nat Genet.

[105] Kwak JW, Laskowski J, Li HY, McSharry MV, Sippel TR, Bullock BL, Johnson AM, Poczobutt JM, Neuwelt AJ, Malkoski SP, et al. Complement activation via a C3a receptor pathway alters CD4+ T lymphocytes and mediates lung cancer progression. Cancer Res. 2018;78:143–56. 10.1158/0008-5472.CAN-17-0240 PMC5810934 29118090

[106] Li HY, McSharry M, Bullock B, Nguyen TT, Kwak J, Poczobutt JM, Sippel TR, Heasley LE, Weiser-Evans MC, Clambey ET, Nemenoff RA (2017). The Tumor Microenvironment Regulates Sensitivity of Murine Lung Tumors to PD-1/PD-L1 Antibody Blockade. Cancer Immunol Res.

[107] Oweida A, Lennon S, Calame D, Korpela S, Bhatia S, Sharma J, Graham C, Binder D, Serkova N, Raben D (2017). Ionizing radiation sensitizes tumors to PD-L1 immune checkpoint blockade in orthotopic murine head and neck squamous cell carcinoma. Oncoimmunology.

[108] Maddalo D, Manchado E, Concepcion CP, Bonetti C, Vidigal JA, Han YC, Ogrodowski P, Crippa A, Rekhtman N, de Stanchina E (2014). In vivo engineering of oncogenic chromosomal rearrangements with the CRISPR/Cas9 system. Nature.

